# Thermal gradient ring reveals different temperature-dependent behaviors in mice lacking thermosensitive TRP channels

**DOI:** 10.1186/s12576-022-00835-3

**Published:** 2022-05-26

**Authors:** Tomoyo Ujisawa, Sachiko Sasajima, Makiko Kashio, Makoto Tominaga

**Affiliations:** 1 Division of Cell Signaling, National Institute for Physiological Sciences, 5-1 Higashiyama, Myodaiji-cho, Okazaki, Aichi 444-8787 Japan; 2 Thermal Biology Group, Exploratory Research Center On Life and Living Systems (ExCELLS), 5-1 Higashiyama, Myodaiji-cho, Okazaki, Aichi 444-8787 Japan; 3 Division of Diabetes, Department of Internal Medicine, Aichi Medical University School of Medicine, 1-1 Yazakokarimata, Nagakute, Aichi 480-1195 Japan; 4 Department of Physiological Sciences, Sokendai (The Graduate University for Advanced Studies), Okazaki, Aichi 444-8787 Japan

**Keywords:** Temperature, Mouse, Thermal gradient ring, Preference, Avoidance

## Abstract

Transient receptor potential (TRP) channels are known as temperature receptors. Each channel has an activation temperature in vitro within the physiological temperature range. Mice deficient in specific TRP channels show abnormal thermal behaviors. However, the role of TRP channels in mouse thermal behavior is not fully understood. We measured thermal behavior using a new type of thermal gradient system, where mice can freely move around the ring floor, thereby avoiding the stereotypical habit that mice have of staying in a corner, as occurs in a rectangular system. With this system, we can also analyze various factors, such as “Spent time,” “Travel distance,” “Moving speed,” and “Acceleration,” to provide more accurate information about mouse behaviors. Further analysis using this system would lead to a better understanding of the molecular basis of thermal behaviors in mice, which could help us develop ways of making humans comfortable in different temperature conditions.

**Supplementary Information:**

The online version contains supplementary material available at 10.1186/s12576-022-00835-3.

## Background

Ambient temperature is received by the skin and sensory neurons, and the information is transmitted to the central nervous system, which leads to various autonomous and non-autonomous responses that control body temperatures. The thermoregulatory system works in central neural networks. When sensory information is relayed to the cerebral cortex through the thalamus, this temperature information is perceived and discriminated [1]. However, this pathway does not lead to thermal behaviors. Another pathway from the spinal cord to the lateral parabrachial nucleus (LPB) and preoptic area (POA) mediates the generation of thermal behaviors, and this occurs before the body core temperature is impacted by the thermal challenge [2]. This spinal–LPB–POA pathway also leads to autonomic heat-gain responses, such as shivering thermogenesis and cutaneous vasoconstriction. Warmth-activated dorsal LPB neurons transmit signals to the median POA (MnPO). This signal activates GABAergic projection neurons in the POA. The activated GABAergic neurons inhibit excitatory pathways that drive sympathetic thermoregulatory effectors, leading to reduced thermogenesis and cutaneous vasodilation. Conversely, cold-activated LPB neurons provide glutamatergic excitation to GABAergic interneurons (rather than to projection neurons) in the MnPO, which reduces the activity of the inhibitory projection neurons in POA neurons. This cold-activated pathway leads to attenuation of cutaneous vasodilation and an increase in heat production.

Transient receptor potential (TRP) channels are sensors for various kinds of chemical and physical stimuli, and some of them, called thermosensitive TRP channels, are activated by temperature changes [3–5]. Currently, eleven thermosensitive TRP channels have been described in mammals, and the temperature activation thresholds of these channels have been determined using electrophysiological techniques. Some thermosensitive TRP channels are expressed in sensory nerve endings or in skin keratinocytes, where ambient temperature is perceived [6, 7]. Although TRPM2 is expressed in peripheral sensory neurons [8], it is also expressed in warmth-sensitive neurons of the POA and contributes to body temperature regulation—namely, TRPM2 is involved in monitoring brain temperature and maintaining normal core body temperature [9]. Nonetheless, TRPM2^−/−^ mice showed normal core body temperatures under healthy conditions.

Many reports showed that thermal behaviors in mice lacking thermosensitive TRP channels differ from those of wild-type (WT) mice. In a linear thermal gradient system or a two-plate test, TRPM8^−/−^ or TRPV3^−/−^ mice were reported to show temperature preferences for the cooler side compared to WT mice. [8, 10–12]. These mutant mice were also shown to stay in the coldest zones (below 16 ℃) for longer than the WT mice. On the other hand, TRPV4^−/−^ mice were reported to show a greater temperature preference for the warmer side compared to WT mice [13]. TRPV1^−/−^ mice showed similar temperature preferences as WT mice at temperatures below 50 ℃ [14], even though the temperature thresholds for heat-evoked activation of TRPV1 are about 43 ℃ in vitro. However, TRPV1^−/−^ mice did show less sensitivity to temperatures over 52 ℃ on a hot plate test [15]. In addition, TRPM2^−/−^ mice do not recognize plate temperature differences between 33 ℃ and 38 ℃ [8]. Temperature sensitivity of TRPA1 is controversial [6, 16]. Mouse TRPA1 was initially reported to be activated by cold temperatures lower than 17 ℃, which is in the noxious range. [17] However, two reports with TRPA1^−/−^ mice showed different results; there was no difference in cold sensitivity between WT and TRPA1^−/−^ mice in one report [18] and the other report showed reduced cold sensitivity in TRPA1^−/−^ mice [19]. Furthermore, TRPA1 has recently been reported to be involved in the acute noxious heat sensing, together with TRPV1 and TRPM3 [20]. Namely, TRPV1/TRPA1/TRPM3 triple knockout mice did not respond to noxious heat stimuli, while responses persisted in single knockout mice. For warmth perception, expression of TRPM8, a cold-sensitive TRP channel, is required [21]. Warmth perception in mouse forepaws is regulated by C-fibers that are either activated or inhibited by warmth. TRPM8^−/−^ mice lack warmth-inhibited C-fibers and did not respond to a temperature increase from 32 ℃ to 42 ℃, suggesting that signaling from warmth-activated C-fibers alone is not sufficient for warmth perception.

Results from previous studies examining thermal behaviors have several problems. For example, the temperature resolution was not high, especially with the two-plate test. In addition, the two-plate test might stress mice more than the Thermal Gradient Ring, as mice can move freely on the ring. Furthermore, a linear thermal gradient system cannot exclude the mouse’s preference to stay in the corner. For these reasons, the Thermal Gradient Ring system was introduced to overcome these disadvantages. One group reported thermal behaviors in mice using the Thermal Gradient Ring system [22]. They used TRPA1^−/−^, TRPM8^−/−^, or double knockout mice to study cold avoidance behaviors. They found that double knockout mice were more deficient in cold avoidance than TRPM8^−/−^ mice. However, they focused on only Spent time to analyze the avoidance. They also combined data from several temperature zones without taking advantage of the high temperature resolution of this system. It may be possible to observe clear temperature preference or avoidance behaviors by analyzing different temperature zones.

These conditions prompted us to make a new analysis with multiple parameters using various TRP-deficient mice. We studied 6 types of mice lacking TRP channels: TRPA1, TRPM2, TRPM8, TRPV1, TRPV3, and TRPV4. In this study, we used the Thermal Gradient Ring system, which has a duplicated temperature gradient on each half of the ring. This apparatus gives us more accurate behavior results, because mice can readily pass through any zone and do not have an opportunity to hide anywhere. Previous thermal behaviors in mice focused mainly on Spent time in each temperature zone. From these analyses, several questions remain regarding the relationships between TRP channels and thermal behaviors. In this study, we sought to analyze multiple parameters of thermal behaviors, including Travel distance and Moving speed.

## Methods

### Animals

We used adult C57BL/6NCr WT or TRP channel-deficient mice that were maintained on a C57BL6/NCr background. Mice lacking *Trpv1, Trpv3, Trpv4, Trpm2, Trpm8,* or *TrpA1* were from David Julius (UCSF) [15], Ardem Patapoutian (Scripps Institute) [10], Makoto Suzuki (Jichi Medical University) [23], Yasuo Mori (Kyoto University) [24], Ardem Patapoutian (Scripps Institute) [11] and David Julius (UCSF) [18], respectively. Ten to sixteen-week-old male mice were housed for at least 1 week before behavioral assays. Ambient temperature was maintained at 24 ± 1 ℃ with lights on from 8:00 to 20:00. All animal care and experimental procedures were approved by our Institutional Animal Care and Use Committee. National Institutes of Health and National Institute for Physiological Sciences guidelines (21A008) were followed and the work was carried out in compliance with ARRIVE guidelines.

### Thermal gradient apparatus

**Fig. 1:**
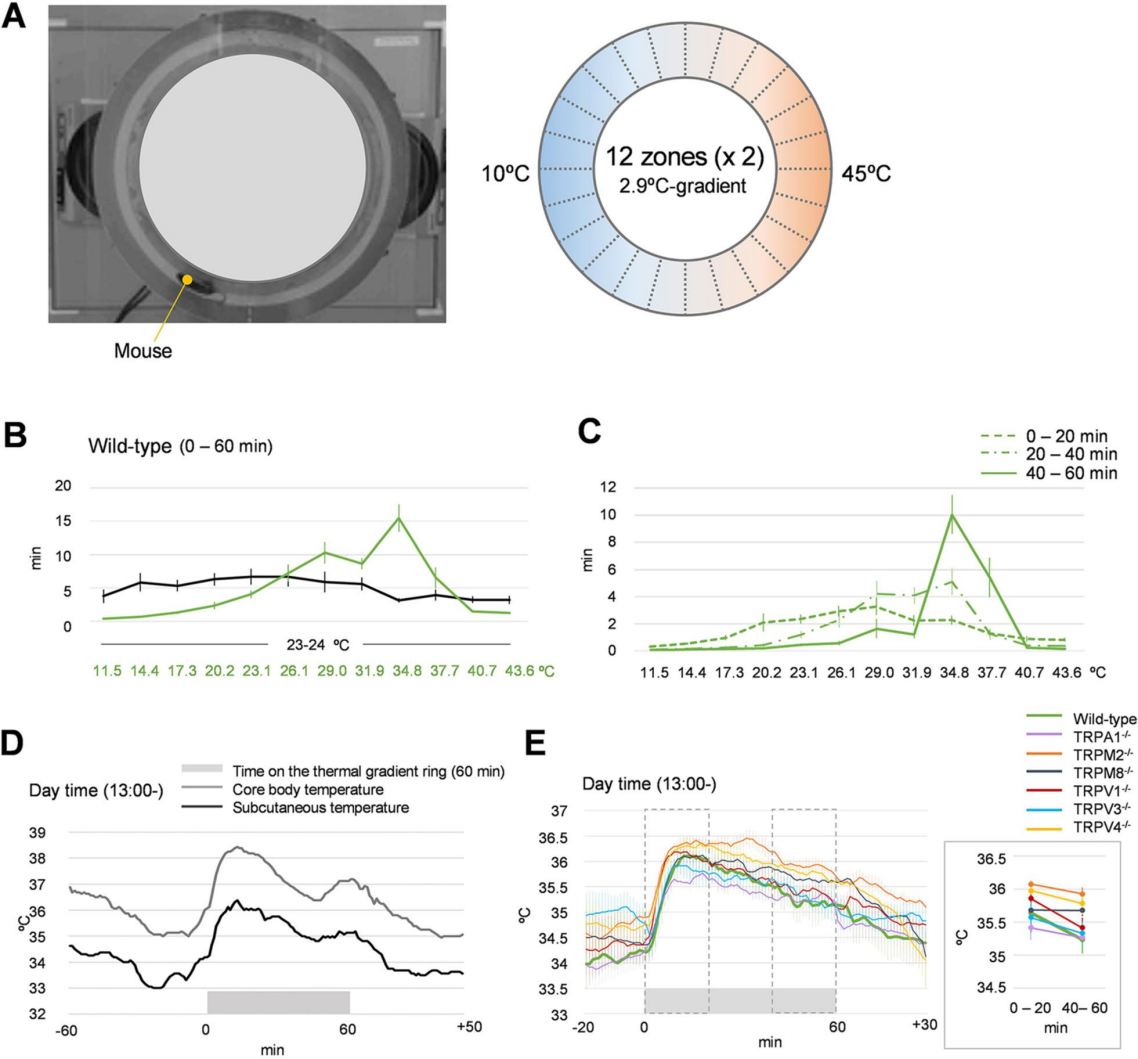
Thermal behaviors on the ring-shaped thermal gradient system. **A** Top camera image of the Thermal Gradient Ring (left) and a schema with the temperature gradient zones marked by different colors (right). There are two temperature control devices under the ring that create a thermal gradient on the floor. There are 12 zones on one half of the ring, and duplicates of the same gradient and zones on the other side. **B** “Spent time” of wild-type mice in the presence (11.5–43.6 ℃, green, *n* = 15) or absence (black, *n* = 15) of a thermal gradient across a 60-min experiment. **C** “Spent time” of wild-type mice on the thermal gradient in 20-min blocks. **D** Transition of subcutaneous (black) and core (gray) body temperatures before, during (a gray bar), and after a thermal behavior test conducted in the daytime (13:00–18:00). **E** Transition of subcutaneous temperatures before, during (a gray bar), and after a thermal behavior test in wild-type mice and mice lacking TRP channels during the daytime (13:00–18:00) (*n* = 3/genotype). Averaged subcutaneous temperatures of all genotypes in the first and last 20 min are shown in the gray box

The Thermal Gradient Ring consists of a 45-cm inner diameter and a 57-cm outer diameter, with a 12-cm height (35560-F, Ugo Basile SRL, Italy). The camera (C920, Logicool, Switzerland) was placed on the upper side of the apparatus. The inner wall of the apparatus was clear, to avoid any camera blind spots. We put a clear board on the top of the apparatus to prevent mice from running away. We used some parameters according to the purpose. For temperature ranges from 10 ℃ to 45 ℃, we set the cooling device to 10 ℃ and the warming device to 45 ℃. Under this condition (10–45 ℃), there was a Δ2.9 ℃ temperature gradient (Fig. 1A). The floor temperature of each graph we showed was recorded in the middle of each zone. Therefore, the middle of the coldest or warmest zone was marked as 11.5 ℃ or 43.6 ℃, respectively. In the setup with a range from 19 ℃ to 36 ℃, there was Δ1.4 ℃ temperature gradient, and the coldest or warmest zone was marked as 19.7 ℃ or 35.3 ℃, respectively. To generate a noxious cold temperature condition, we created a range from 6 ℃ to 49 ℃ (a temperature gradient of about Δ3.5 ℃). In this case, the middle of the coldest zone was 7.8 ℃. In addition, this new Thermal Gradient Ring had an infrared camera and an infrared transmission inner wall, which was provided from Ugo Basile Srl, so that we could monitor mouse behaviors in the dark. The apparatus was further equipped with a floor temperature monitoring system, so that mouse body temperature could be measured.

### Behavioral measurements

Behavioral assays were performed between 10:00 and 20:00. The day before the thermal gradient test, each mouse was acclimated for 30 min in the thermal gradient apparatus, while its floor was at room temperature (23–24 ℃). The stability of the gradient was monitored each day using a thermometer (HFT-51, ANRITSU, Japan). Each mouse was placed in the temperature gradient apparatus individually. Behavioral data were collected by videotaping for 60 min and were automatically analyzed for “Spent time,” “Travel distance,” or “Moving speed” by ANY-maze software (Stoelting Co.). The ANY-Maze software recognizes speed when the mouse moves, but no speed is calculated when the mouse does not move. Moving speed was calculated about every 0.1 s with ANY-maze software. We then used this data to calculate ‘‘Acceleration” with the following equation: m/s^2^ = [v(a)–v(b)]/[t(a)–t(b)]. The (b) refers to the time point before the (a) time point. The v and t indicate speed and time position, respectively.

### Body temperature measurements

We inserted a temperature logger (nano tag, ACOS, Nagano, Japan) to measure body temperature in the intraperitoneal cavity or subcutaneously in the back. After surgery, mice were allowed to recover for 1 week prior to any behavioral testing. The logger was controlled by Felica (RC-S380, SONY, Japan) to start or finish the measurement. The results were loaded using Felica and monitored by the nano tag viewer software. We began recording measurements before the behavior test. A mouse was housed for 30 min and transferred to the apparatus for a duration of 60 min. After the test, the mouse was housed for 30 min again, and we stopped recording the temperature.

### Statistics

The results of behavioral assays were analyzed using Welch’s *t* test or a one-way ANOVA with Tukey correction using OriginPro 2020b (OriginLab Corporation, Northampton, MA, USA). Error bars in the figures represent standard errors of the mean.

## Results

### Thermal behaviors in mice using a Thermal Gradient Ring system

We analyzed thermal behaviors in mice using a Thermal Gradient Ring system in which the ring floor was divided into 24 zones from cold to hot, with the same temperatures occurring in two zones, allowing mice to freely move based on their temperature preference (Fig. 1A). We first set temperatures from 10 ℃ to 45 ℃ in a room with an ambient temperature of 23 ℃ to 24 ℃. Each floor zone had a Δ2.9 ℃-gradient from 11.5 ℃ to 43.6 ℃. We placed mice into this system for 60 min and continuously observed mouse movements. When there was no thermal gradient (i.e., when the floor temperature was the same as room temperature), WT mice moved equally between zones. On the other hand, when there was a temperature gradient, WT mice were more likely to stay at specific temperature zones, indicating a preference (Fig. 1B). Interestingly, WT mice did not show obvious temperature preferences in the first 20 min of the experiment, likely because the mice were first exploring their preferred temperatures. Eventually, the mice chose specific temperatures to stay at for the last 20 min of the experiment, and the preferred temperature was around 35 ℃ (Fig. 1C).

Mice are nocturnal animals and move more at night, at which time they also have higher body temperatures. Therefore, to examine the effects of body temperature on thermal behaviors, we put a small logger to measure the mouse’s body temperature [25]. The loggers were placed subcutaneously in the back or in the intraperitoneal space (core body temperature). We observed circadian changes in both the subcutaneous and core temperatures, where temperatures were high at night and lower during the day, as previously reported (Additional file 1: Fig. S1A). In addition, subcutaneous temperatures were approximately 2 ℃ lower than core body temperatures. To examine the relationship between body temperature and behaviors in the thermal gradient system, WT mice were first placed on the Thermal Gradient Ring without a thermal gradient. Subcutaneous and core temperatures were measured under this condition for 30 min. Then, the thermal gradient system was turned on (10–45 ℃), and was stabilized within 30 min, at which point we measured the mouse’s movements. Both the subcutaneous and core temperatures became elevated when mice were placed on the ring apparatus, likely because they began exploring, and they were reduced across time (Additional file 1: Fig. S1B). During the daytime (13:00 to 18:00) measurements, mice were placed on a ring, where the temperatures were already set. Both subcutaneous and core temperatures were similarly elevated and reduced (Fig. 1D). Changes in both the subcutaneous and core temperatures were essentially the same at nighttime (21:00-) as in the daytime, except that both temperatures were slightly higher before the behavior assay (Additional file 1: Fig. S1C).

Because both the subcutaneous and core temperatures changed similarly and because the temperature logger measurement in the intraperitoneal space was not affected by the ring floor temperature, we compared only the subcutaneous body temperatures between WT mice and mice lacking TRP channel genes. Subcutaneous body temperatures did not differ significantly between mice examined in the daytime (Fig. 1E) and nighttime (Additional file 1: Fig. S1D). Based on these results, we concluded that initial body temperatures do not affect body temperature in the thermal behavior assay, and thus decided to conduct all of the following experiments in the daytime (13:00 to 18:00).

### Mice lacking thermosensitive TRP channels showed various temperature preference and avoidance behaviors

**Fig. 2:**
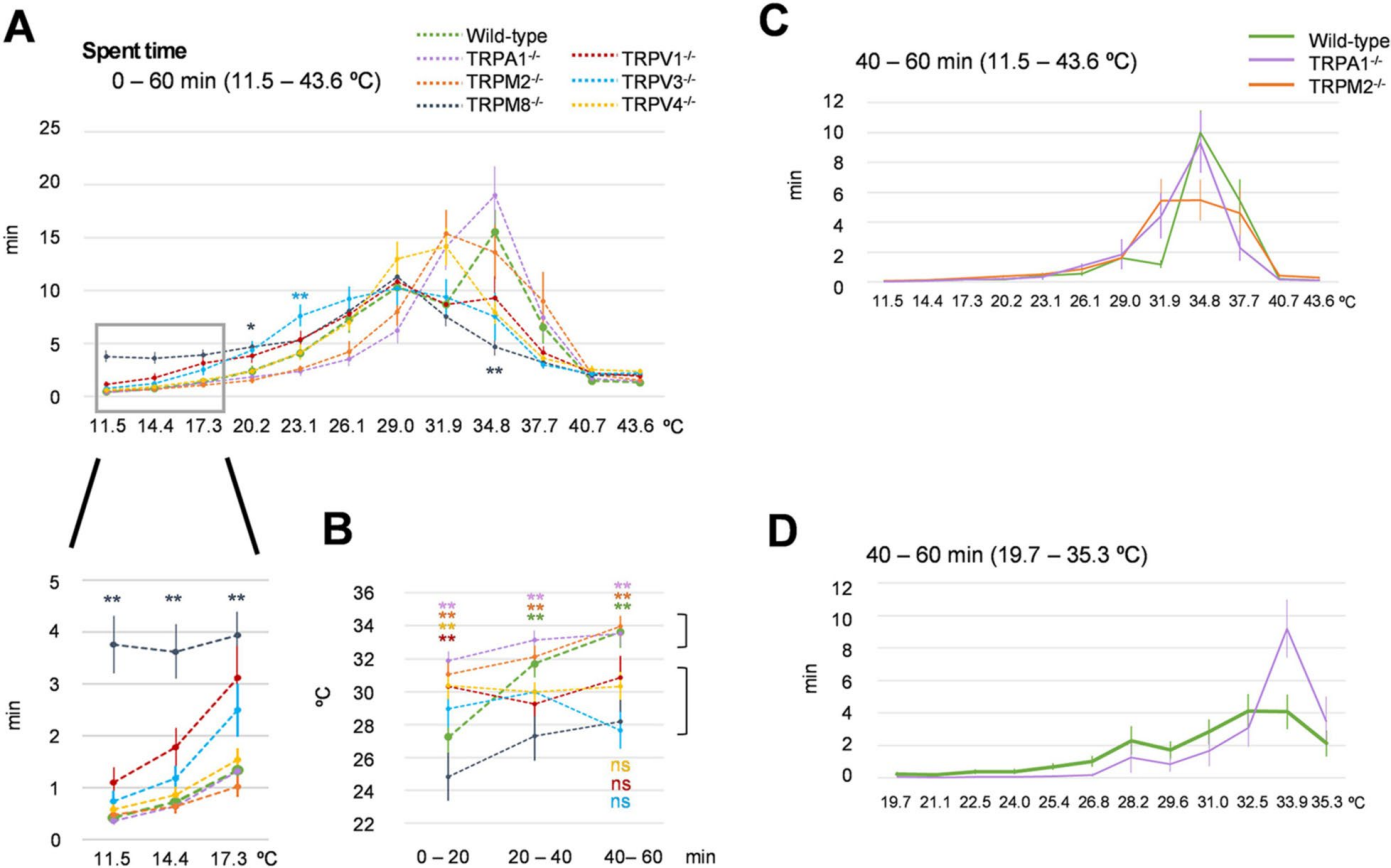
“Spent time” on the thermal gradient ring. **A** “Spent time” of all genotypes at each temperature zone from 11.5 ℃ to 43.6 ℃ over a 60-min period (wild type *n* = 15, TRPA1^−/−^ *n* = 14, TRPM2^−/−^ *n* = 13, TRPAM8^−/−^ *n* = 14, TRPV1^−/−^ *n* = 11, TRPV3^−/−^ *n* = 13, TRPV4^−/−^ *n* = 15). The gray box shows an enlarged view of the lower temperatures. *, **p < 0.05, *p* < 0.01 vs. wild type. **B** Transition of peak “Spent time” temperature in each genotype in the first, middle, and last 20 min of the experiment. The color of each genotype is the same as shown in panel A. Mice were classified into two groups, as shown by the brackets. **C** “Spent time” in each temperature zone of wild-type (green), TRPM2^−/−^ (orange), and TRPA1^−/−^ (purple) mice under the condition of 11.5 ℃ to 43.6 ℃ during the last 20 min (40–60 min). *p* values are shown on the table. **D** “Spent time” in each temperature zone of wild-type (green) and TRPA1^−/−^ (purple) mice under the condition of 19.7 ℃ to 35.3 ℃ during the last 20 min. (wild type *n* = 12, TRPA1^−/−^ *n* = 14, TRPM2^−/−^ *n* = 12, TRPAM8^−/−^ *n* = 12, TRPV1^−/−^ *n* = 11, TRPV3^−/−^ *n* = 13, TRPV4^−/−^ *n* = 15). All error bars represent standard errors of the mean. Colors of asterisks and ns (not significant) indicate the comparison between wild-type mice and mice lacking TRP channels, as shown in panel A. *p < 0.05, **p < 0.01 vs. wild type

There are 11 thermosensitive TRP channels, each with a distinct temperature threshold for activation. [5] However, most thermal behavior data have been reported using the two temperature plate choice assay or the linear thermal gradient assay [10, 11], which might not be suitable for the analysis of temperature preference or avoidance. Therefore, in this study, we analyzed thermal behaviors across a 60-min duration in mice lacking *Trpv1, Trpv3, Trpv4, Trpm2, Trpm8,* or *TrpA1* using the Thermal Gradient Ring system with different behavioral parameters. When we analyzed "Spent time" on the floor with no thermal gradient, all genotypes visited all zones equally and showed no spatial preference or avoidance (Additional Fig. 2A). When we analyzed "Spent time" on the floor with a temperature gradient from 11.5 ℃ to 43.6 ℃, WT mice stayed longer in the 34.8 ℃ zone, as did TRPA1^−/−^ or TRPM2 ^−/−^ mice (Figs. 1 and 2A). Although TRPV4^−/−^ mice have been reported to stay longer in higher temperature zones compared with WT mice over a 2 h period in a linear thermal gradient assay [13], TRPV4^−/−^ mice in our assay stayed primarily in the lower temperature zones (31.9 ℃) compared to WT mice. TRPM8^−/−^ and TRPV3^−/−^ mice stayed longer in the lower temperature zones, as previously reported [10, 11, 14]. Only TRPM8^−/−^ mice showed obvious differences in the time spent at the 11.5 ℃ to 17.3 ℃ zone (Fig. 2B, Additional file 2: Fig. S2B, C), which was expected as TRPM8^−/−^ mice do not express the TRPM8 cold sensor, and, therefore, readily enter the cold zones. We did not observe any difference in temperature preference between WT and TRPA1^−/−^ mice.

We next analyzed the transition of peak temperatures across the 60-min experiment, assessing which temperatures the mice stayed at every 20 min (Fig. 2B, Additional file 2: Fig. S2B). The mice were divided into two groups: one group (TRPM2^−/−^, TRPA1^−/−^) gradually moved to the warm zone over time, similar to the WT mice that stayed longer in the zone of around 34 ℃, and the other group (TRPV1^−/−^, TRPV3^−/−^, TRPV4^−/−^, TRPM8^−/−^) continuously stayed at about 30 ℃. To examine the difference among WT, TRPA1^−/−^, and TRPM2^−/−^ mice, we extracted data during the 40–60 min window, when mice tended to settle at specific temperatures (Fig. 2C). TRPM2^−/−^ mice stayed in wider temperature zones and did not have a specific peak. TRPA1^−/−^ mice showed the same temperature preference as WT mice in this time period. Therefore, we increased the temperature resolution by changing the floor temperature range to 19.7 ℃ to 35.3 ℃ (this was the 19–36 ℃ temperature setting) (Fig. 2D, Additional file 3: Fig. S3). Under this setting, TRPA1^−/−^ mice showed clearer temperature preferences, with a peak of 33.9 ℃ compared with WT mice, and this temperature preference of TRPA1^−/−^ mice was observed as early as in the 40–60 min period (Additional file 3: Fig. S3C).

### Analysis of other parameters: “Travel distance”, “Moving speed,” and “Acceleration”

**Fig. 3:**
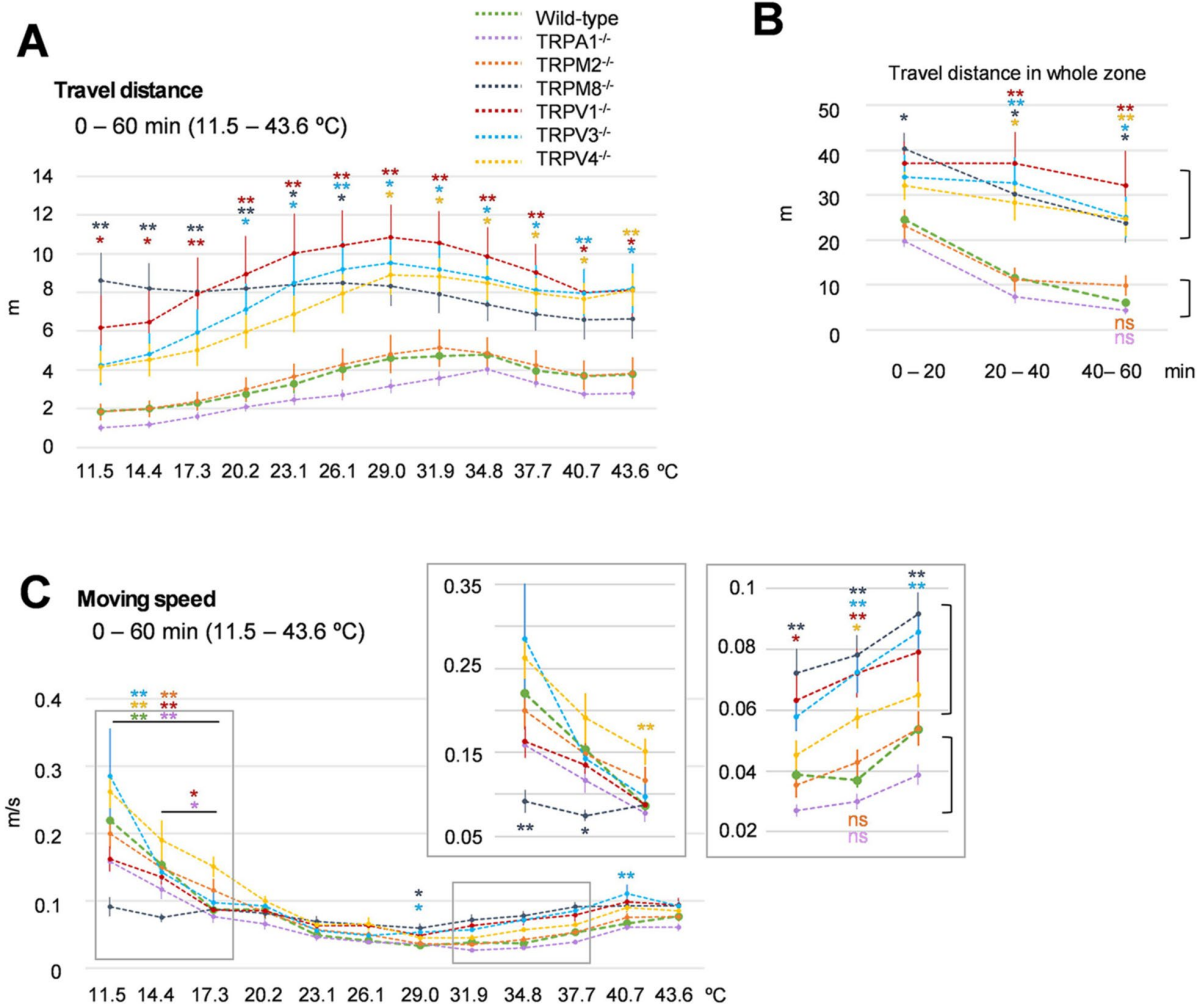
“Travel distance” and “Moving speed” on the Thermal Gradient Ring. **A** “Travel distance” in each temperature zone under the condition of 11.5–43.6 ℃ across a 60-min period (wild type *n* = 15, TRPA1^−/−^ *n* = 14, TRPM2^−/−^ *n* = 13, TRPAM8^−/−^ *n* = 14, TRPV1^−/−^ *n* = 11, TRPV3^−/−^ *n* = 13, TRPV4^−/−^ *n* = 15). **B** Transition of “Travel distance” as shown by whole temperature zones for each genotype in the first, middle, and last 20 min of the experiment. The color of each genotype is the same as shown in panel A. Mice were classified into two groups, as shown by the brackets. **C** “Moving speed” in each temperature zone under the condition of 11.5–43.6 ℃ in a 60-min period. The color of each genotype is the same as shown in panel A. Data from 11.5 ℃ to 17.3 ℃ and from 31.9 ℃ to 37.7 ℃ are enlarged. All error bars represent standard errors of the mean. Colors of asterisks and ns (not significant) indicate the comparison between wild-type mice and mice lacking TRP channels, as shown in panel A. * *p* < 0.05, ** *p* < 0.01 vs. wild type

Next, we analyzed other parameters (Travel distance and Moving speed) in each temperature zone using the 11.5–43.6 ℃ gradient setting. Travel distance was shorter at the low temperatures for all mice except for TRPM8^−/−^ mice, which had a high Travel distance even in the 11.5 ℃ zone (Fig. 3A), likely because they do not sense cold temperatures. We also analyzed Travel distance every 20 min (Fig. 3B). The mice could be divided into two groups: one group (TRPM2^−/−^, TRPA1^−/−^) showed reduced Travel distance across time, which was similar to WT mice, and the other (TRPV1^−/−^, TRPV3^−/−^, TRPV4^−/−^, TRPM8^−/−^) traveled further than WT mice and kept travelling throughout the entire 60-min experiment. Interestingly, this grouping was the same as the one we found for the Spent time analysis (Fig. 2B). This feature was consistent even without the presence of a thermal gradient (Additional file 4: Fig. S4A, B), suggesting that this phenomenon was not related to the floor temperature gradient.

We also analyzed Moving speed in each zone, because mice were expected to move faster in the temperature zones that they want to avoid. Because mice showed similar Spent time at temperature zones less than 20.2 ℃ or over 40.7 ℃, it is unclear whether mice felt uncomfortable when they were in these temperatures (Fig. 2A). We found that TRPM8^−/−^ mice had a consistent Moving speed, even at the coldest zone of 11.5 ℃, while the other genotypes, including WT mice, showed a higher Moving speed in the coldest zone (Fig. 3C, Additional file 5: Fig. S5C). This interpretation could be justified by the result that mice moved in all zones at a speed of less than 0.1 m/s (0.068 m/s) when there is no thermal gradient on the floor (Additional file 5: Fig. S5A). This indicated that mice felt comfortable when moving at a speed of less than 0.1 m/s, and did not change over time (Additional file 5: Fig. S5B). WT, TRPA1^−/−^, and TRPM2^−/−^ mice moved at a slower speed than the other genotypes in the 34.8 ℃ zone, which was consistent with the data that WT, TRPA1^−/−^, and TRPM2^−/−^ mice stayed longest in the temperature zone (Additional file 2: Fig. S2B).

**Fig. 4:**
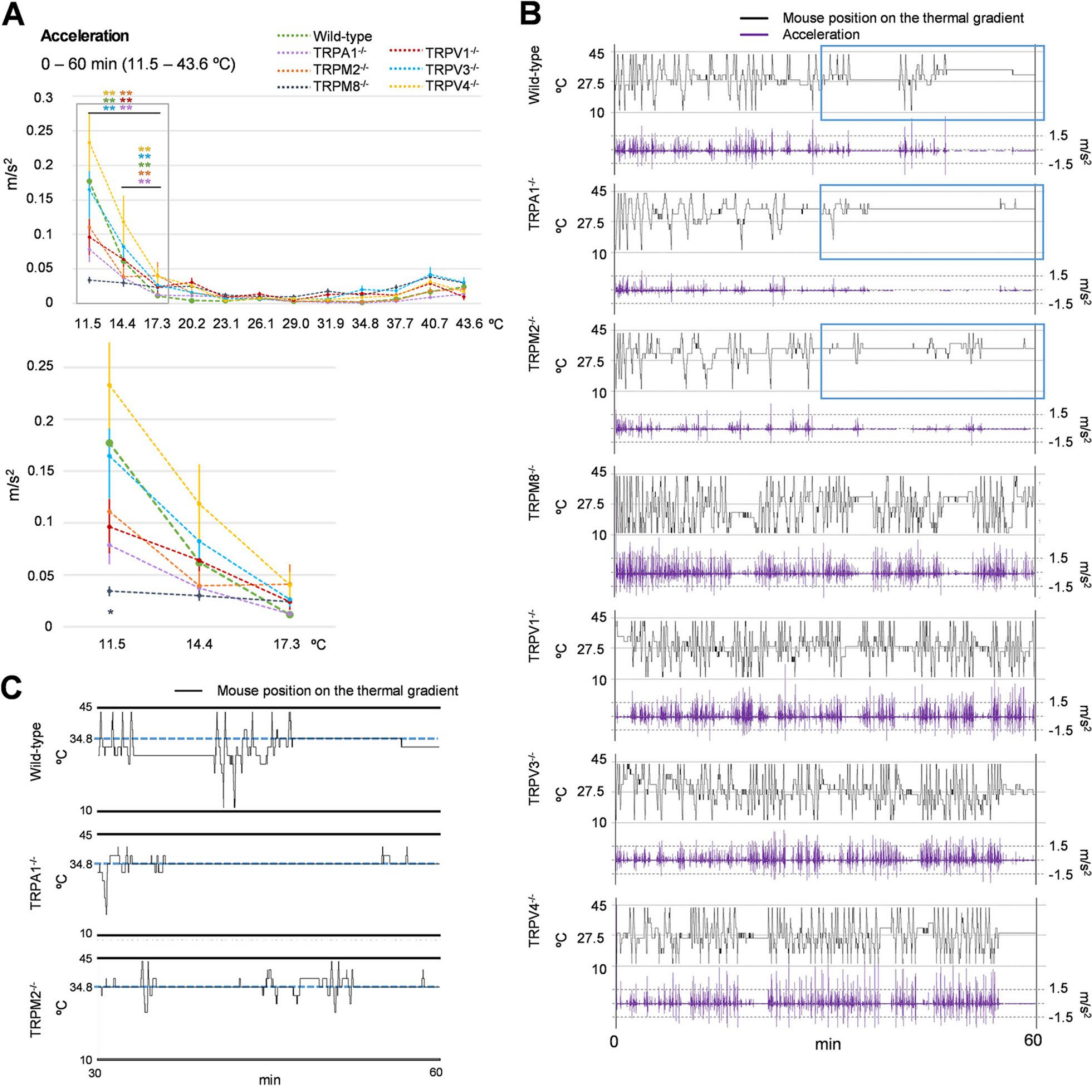
“Acceleration” on the thermal gradient ring. **A** “Acceleration” in each temperature zone under the condition of 11.5–43.6 ℃ across a 60-min period (wild type *n* = 15, TRPA1^−/−^ *n* = 14, TRPM2^−/−^ *n* = 13, TRPAM8^−/−^ *n* = 14, TRPV1^−/−^ *n* = 11, TRPV3^−/−^ *n* = 13, TRPV4^−/−^ *n* = 15). Data from 11.5 ℃ to 17.3 ℃ are enlarged in the grey box. All error bars represent standard errors of the mean. Colors of asterisks indicate the comparison between wild-type mice and mice lacking TRP channels, as shown in panel A. *p < 0.05, **p < 0.01 vs. wild type. **B** Representative tracking data (black) with “Acceleration” (purple) in each genotype under the condition of 11.5–43.6 ℃ across a 60-min experiment. “Acceleration” and deceleration are shown in upward and downward directions, respectively. **C** Expanded tracking data of wild-type, TRPA1^−/−^, and TRPM2^−/−^ mice in the last 30 min of the experiment (30–60 min) in panel B

Moving speed could be a factor of Travel distance, possibly revealing that mice that travelled a lot move faster compared to mice that travel less. Therefore, to exclude any effects by Travel distance we analyzed the mean of Acceleration to find the temperature zone at which mice changed their Moving speed. There was no difference in mean of Acceleration between WT mice and mice lacking TRP channels in the warm temperature zones. All mice showed an increase in Acceleration in the temperature zones of 11.5 ℃ and 14.4 ℃, except for TRPM8^−/−^ mice, which did not accelerate in any zone (Fig. 4A). We also analyzed the real tracking data (black lines) with Acceleration (purple lines) across all temperature zones. Since the Thermal Gradient Ring is a circular device, Acceleration can be calculated as both positive and negative values depending on whether the mouse is running or turning back, respectively. Changes in moving was more clearly recognized when we plotted the temperature at which mice stayed and Acceleration as the y axis and time (0–60 min) as the *x*-axis (Fig. 4B). WT, TRPA1^−/−^, and TRPM2^−/−^ mice stopped moving at temperatures around 35 ℃ in the midpoint of the analysis (Fig. 4C), while TRPM8^−/−^, TRPV1^−/−^, TRPV3^−/−^, and TRPV4^−/−^ mice continued to move.

### Analysis in the cold and hot temperature zones

**Fig. 5:**
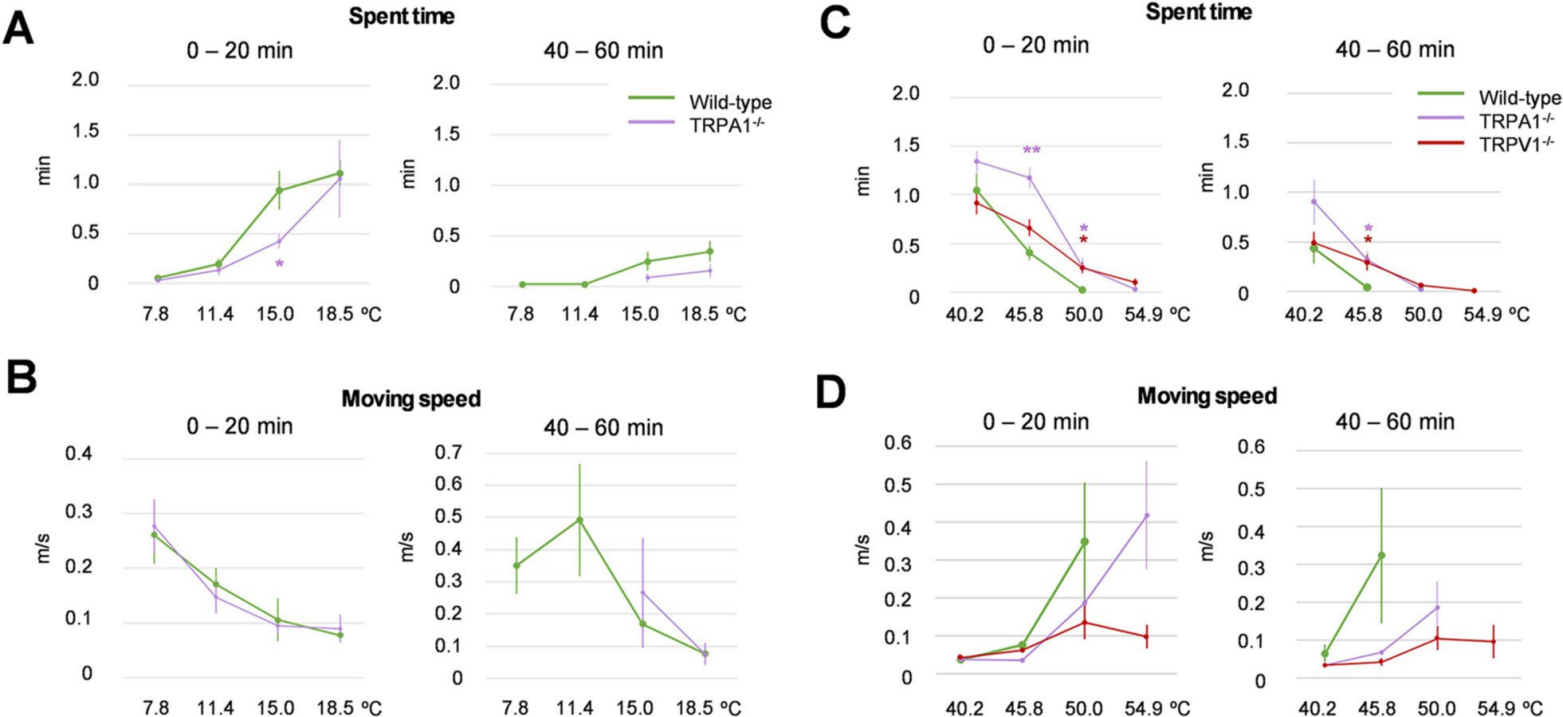
Mouse behaviors at noxious cold or hot temperatures. **A** “Spent time” in the cold temperatures (7.8–18.5 ℃) for wild-type (green, *n* = 13) and TRPA1^−/−^ (purple, *n* = 13) mice under the condition of 7.8–47.2 ℃ in the first 20 min (0–20 min) and the last 20 min (40–60 min) of the experiment. *p < 0.05. **B** “Moving speed” at the cold temperatures (7.8–18.5 ℃) for wild type (green, *n* = 13) and TRPA1^−/−^ (purple, *n* = 13) under the condition of 7.8–47.2 ℃ during the first 20 min (0–20 min) and the last 20 min (40–60 min) of the experiment. **C** “Spent time” in the hot temperatures (40.2–54.9 ℃) for wild type (green, *n* = 11), TRPA1^−/−^ (purple, *n* = 10), and TRPV1^−/−^ (red, *n* = 11) under the condition of 14.6 ℃ to 54.9 ℃ in the first 20 min (0–20 min) and the last 20 min (40–60 min) of the experiment. *p < 0.05 vs. wild type. **D** “Moving speed” in hot temperatures (40.2–54.9 ℃) for wild type (green, *n* = 11), TRPA1^−/−^ (purple, *n* = 10), and TRPV1^−/−^ (red, *n* = 11) under the condition of 14.6–54.9 ℃ in the first 20 min (0–20 min) and the last 20 min (40–60 min) of the experiment. Error bars represent standard errors of the mean

TRPA1 was initially reported as a cold sensor under 17 ℃, [17] although there is controversy surrounding this [6, 16]. Therefore, we examined mouse behaviors under even colder conditions (7.8–47.2 ℃, which were achieved using the temperature setting of 5.0–54.9 ℃). However, WT and TRPA1^−/−^ mice did not stay long at temperature zones lower than 11.4 ℃ in the first 20 min, and there was no difference in Spent time and Moving speed under this gradient setting (Fig. 5A, B). WT mice still entered the temperature zones lower than 11.4 ℃, although Spent time was decreased in these zones in the last 20 min (40–60 min). On the other hand, TRPA1^−/−^ mice did not enter these cold zones during the last 20 min. Consistent with the Spent time results, WT mice showed increased Moving speed both in the first and last 20 min, which was similar to TRPA1^−/−^ mice. Recently, TRPM3/TRPV1/TRPA1-triple knockout mice were reported to show an absence of responses to noxious heat stimuli [20]. Therefore, we examined mouse behaviors in conditions over 40 ℃, with the thermal gradient being 14.6 ℃ to 54.9 ℃ (achieved by setting the temperature from 10.0 to 60.0 ℃). TRPV1 is a sensor for noxious heat, and TRPV1^−/−^ mice are known to be insensitive to temperatures over 52 ℃ on a hot plate test [15]. WT mice did not enter temperature zones over 50 ℃, although both TRPA1^−/−^ and TRPV1^−/−^ mice stayed in the high temperature zones in the first 20 min (Fig. 5C). However, TRPA1^−/−^ mice did not enter this zone in the last 20 min, suggesting that TRPV1^−/−^, but not TRPA1^−/−^ tolerate the noxious high temperatures. These phenotypes were also recognized by the slower Moving speed of TRPV1^−/−^ mice than WT mice in the high temperature zones (Fig. 5D).

## Discussion

In this study we characterized temperature-dependent behaviors of mice lacking thermosensitive TRP channels. We used a ring-shaped temperature gradient system to accurately measure values for Spent time or Moving speed, as mice are able to readily traverse all the temperature zones in this apparatus. Many thermal behaviors in thermosensitive TRP channel-deficient mice have been reported using linear-shaped apparatuses [10, 11]. However, a stereotypical mouse behavior is to hide in the corner, which introduces a bias when analyzing mouse behaviors, where the cold or hot zones are positioned at the corner end. In this study, we overcame this disadvantage with a ring-shaped apparatus. Moreover, we provided a detailed characterization of mouse thermal behaviors by measuring several other parameters, such as Travel distance, Moving speed, and Acceleration. From the Spent time result alone, it was difficult to conclude which temperature mice felt comfortable in, but it became clear when we examined other parameters, such as Moving speed. From the mean of Acceleration data, we found that all genotypes, except TRPM8^−/−^ mice, had an accelerated Moving speed at temperatures below 14.4 ℃, suggesting that those mice recognized noxious cold temperatures (Fig. 4A).

Conversely, TRPM8^−/−^ mice showed no evasive behaviors in the low temperature zones.

In one previous report using a cold plate assay, TRPA1^−/−^ mice showed fewer paw lifts at 0 ℃ than WT mice [19], but another report showed no difference between WT and TRPA1^−/−^ mice [18]. Previously, in ring gradient assay, authors reported that TRPA1^−/−^ mice showed similar Spent time in all zones as WT mice, but they quickly recognized warmer zones as preferable [22]. Our results also showed that only TRPA1^−/−^ mice showed a preference for 33.9 ℃ as early as 20–40 min in a thermal gradient of 19.7 ℃ to 35.3 ℃ (Additional file 3: Fig. S3), while clear temperature preferences were recognized in all genotypes when using a thermal gradient of 11.5 ℃ to 43.6 ℃ (Additional file 2: Fig. S2B). TRPA1^−/−^ mice did not enter either the cold (below 11.4 ℃) or hot (54.9 ℃) temperature zones, indicating that TRPA1^−/−^ mice avoided noxious temperatures. These results suggest that TRPA1^−/−^ mice have a high temperature sensitivity.

TRPM2^-/-^ mice were previously reported to lose a preference when presented a choice between 33 ℃ and 38 ℃ in the two-plate assay, but when offered plates of 33 ℃ or 28 ℃, they spent longer at 33 ℃ like WT mice [8]. We found that TRPM2^-/-^ mice spent similar amounts of time in the 31.9 ℃ to 37.7 ℃ zones, whereas WT mice preferred the 34.8 ℃ zone over the 31.9 ℃ or 37.7 ℃ zones (Fig. 2C). These data suggest that TRPM2^-/-^ mice do not recognize temperature differences between 31.9 ℃ to 37.7 ℃, which is consistent with the results of a previous study [8]. Although TRPM2 was also reported to detect warm brain temperature that in turn induces a reduction in body temperature [9, 26], whether TRPM2 expressed in the hypothalamus is involved in this behavioral phenomenon is unclear.

TRPV1^−/−^ mice exhibited longer latencies for paw withdrawal responses to temperatures over 52.5 ℃ in the hot plate test. [15] Another report using a linear temperature gradient assay showed that TRPV1^−/−^ mice spent longer in the 42 ℃ to 55 ℃ zones than WT mice, although the same report showed a difference in the hot plate assay in temperature ranges over 52 ℃ [14]. Our results are similar to the latter report using a linear temperature gradient assay. TRPV1^−/−^, but not WT mice, entered the zone of 50.0 ℃ during the 40–60 min mark of the experiment with a thermal gradient of 14.6 ℃ to 54.9 ℃ (Fig. 5C, D). These results could correspond to TRPV1 being act1ivated by high temperatures over 43 ℃ in vitro. [27, 28].

TRPM8^−/−^ mice were reported to have no temperature preferences between 15 ℃ and 30 ℃ in a previous study using a two-plate assay [12]. TRPM8^−/−^ mice were also reported to show a wider temperature preference than WT mice in the 23.0 ℃ to 34.0 ℃ zones in a linear gradient assay from 15.0 to 53.5 ℃. [11] These results are similar to our results. However, in the linear gradient assay, the behaviors of TRPM8^−/−^ mice did not differ from WT mice in temperature zones below 21.5 ℃. In our study, TRPM8^−/−^ mice spent most of their time in the 29.0 ℃ zone, and spent more time in temperature zones below 20.2 ℃ compared to WT mice (enlarged inset in Fig. 2A, Additional file 2: Fig. S2C). In addition, TRPM8^−/−^ mice travelled equally across all zones, without any change in their Moving speed (Fig. 3A, C). Furthermore, TRPM8^−/−^ mice stayed longer and moved slower than other genotypes in zones below 14.4 ℃ (Figs. 2A, 3C), which could indicate that TRPM8^−/−^ mice did not show avoidance behaviors at temperatures from 11.5 ℃ to 43.6 ℃, corresponding to the TRPM8 temperature threshold of 26 ℃ to 28 ℃ determined in vitro. These data indicate that a detailed temperature preference can be analyzed with the Thermal Gradient Ring assay system.

TRPV3^−/−^ mice were reported to spend more time in temperatures ranging from 20.5 ℃ to 24.5 ℃ in a previous study that used a linear gradient assay from 15 ℃ to 55º C [10], and these mice have broad temperature preferences, which aligns with our results. However, in the linear gradient assay, TRPV3^−/−^ mice showed significantly longer time spent in the coldest zone of 15 ℃ compared to WT mice [10], which was not observed in our ring gradient assay. Given that TRPV3 channels are activated by warm temperatures in vitro, the apparent cold preference observed in TRPV3^−/−^ mice may be due to the stereotypical behavior of mice preferring to stay in a corner.

As mentioned before, TRPV4^−/−^ mice preferentially stayed in zones from 29.0 ℃ to 31.9 ℃ (Fig. 2A), which was similar to data previously reported from a linear gradient assay [13]. However, there was a difference in the temperature preference of WT mice between our study and the linear gradient assay, in which WT mice showed a temperature preference of around 28.1 ℃. Although we cannot explain the difference in WT mice between the two studies, a difference in the housing environment could lead to differences in results. Alternatively, WT mice behaviors could be more precisely analyzed in the Thermal Gradient Ring assay.

All thermosensitive TRP channels have temperature thresholds for their activation, as determined in vitro. However, it is difficult to see relationships between the reported temperature thresholds and thermal behaviors. For example, the activation temperature thresholds for TRPV3 and TRPV4 are over 32–39 ℃ and 27–35 ℃, respectively. TRPV3^−/−^ mice showed significantly more Spent time in the 23.1 ℃ zone, which is far from the temperature range activating TRPV3. TRPV4^−/−^ mice preferred to stay in zones from 29.0 ℃ to 31.9 ℃, which is slightly cooler than the preference of WT mice.

In this study, we found two groups of mice. One group (WT, TRPA1^−/−^, TRPM2^−/−^ mice) showed a peak of Spent time at about 34 ℃ and these mice traveled less, and the other group (TRPV1^−/−^, TRPV3^−/−^, TRPV4^−/−^, TRPM8^−/−^ mice) showed a peak of Spent time at about 28 ℃ to 30 ℃ and travelled more (Figs. 2B, 3B). This grouping based on Travel distance was more clearly recognized when we plotted temperatures at which mice stayed and Acceleration as the y axis, and time (0–60 min) as the x axis (Fig. 4B), and it was more clearly observed at thermal gradients of 11.5 ℃ to 43.6 ℃ and 19.7 ℃ to 35.3 ℃ (Fig. 3B, Additional file 4: Fig. S4C). In addition, the difference in Travel distance was not related to the thermal gradient, because these two groups were observed when no thermal gradient was used (Additional file 4: Fig. S4A). Furthermore, we did not find body temperature differences among genotypes during the behavior test (Fig. 1E), indicating that less Travel distance was not related to body temperature. These data suggest that the difference in Travel distance is an endogenous property of mice, regardless of their thermosensing ability. However, the grouping of Travel distance coincided with the grouping of the temperature at which mice spent most of their time. Although this is an interesting phenomenon, we currently cannot explain the apparent relationship between Travel distance and peak of Spent time, and further experiments are needed to clarify this. In our knockout mice, the thermosensitive TRP channels we studied were ablated in the whole body, including the brain, sensory neurons, and skin keratinocytes, which may partially explain the phenomena observed, especially the difference in Travel distance, which was also observed without a thermal gradient.

TRPM8^−/−^ mice showed peak Spent time in cooler temperature zones. This result could be simply interpreted as the TRPM8^−/−^ mice do not recognize the cool temperatures that WT mice avoid. However, one report showed that TRPM8 was necessary for warmth perception [21]. Warmth perception was suggested to require signals not only from warmth-activated C-fibers, but also from warmth-inhibited C-fibers expressing TRPM8. TRPM8^−/−^ mice have impaired warmth perception and have different peak temperatures for Spent time compared to WT mice. More experiments are thus needed to understand the molecular mechanisms underlying warmth perception.

TRPC5 was recently reported to be involved in noxious cold sensation in mouse odontoblasts [29], although in this study we did not examine the contribution of TRPC5 to mouse behaviors as we lacked TRPC5-deficient mice. Examining the involvement of TRPC5-deficient mice with the Thermal Gradient Ring would be intriguing, especially if TRPC5 expression in the sensory neurons could be established.

In conclusion, the Thermal Gradient Ring assay revealed several interesting phenomena regarding temperature-dependent behaviors of mice. Further analysis using this system would lead to a better understanding of the molecular basis of thermal behaviors in mice, which could be helpful to developing ways to make humans comfortable in different temperature conditions.

### Supplementary Information

**Additional file 1: Figure S1**. Changes in body temperatures. (A) Transition of subcutaneous (black) and core (gray) temperatures of WT mice in three consecutive days in the home cage. Nighttime (when the light is turned off from 20:00 to 8:00) is indicated by gray bars. (B) Transition of subcutaneous (black) and core (gray) temperatures of WT mice on the Thermal Gradient Ring during the daytime (13:00–18:00). The Thermal Gradient Ring system is turned on at the 30-min mark of the mouse being placed in the apparatus and the ring floor temperatures are considered stable at 60 to 120 min (shown in gray). (C) Transition of subcutaneous (black) and core (gray) body temperatures before, during (a gray bar), and after a thermal behavior test during the nighttime (21:00–6:00). (D) Transition of subcutaneous temperatures before, during (a gray bar), and after a thermal behavior test in wild-type mice and in mice lacking TRP channels during the nighttime (21:00-) (n=3/genotype). Averaged subcutaneous temperatures of all genotypes in the first and last 20 min are shown in the gray box. Error bars represent standard errors of the mean.

**Additional file 2: Figure S2**. ‘‘Spent time’’ in the condition from 11.5 to 43.6 ℃. (A) “Spent time” for all genotypes without a thermal gradient across a 60-min experiment (wild type n=12, TRPA1-/- n=14, TRPM2-/- n=12, TRPAM8-/- n=12, TRPV1-/- n=11, TRPV3-/- n=13, TRPV4-/- n=12). (B) “Spent time” for all genotypes at each temperature zone from 11.5 ℃ to 43.6 ℃ across 60 min, as broken down by the first (top), middle (middle), and last (bottom) 20 min of the experiment. (wild type n=15, TRPA1-/- n=14, TRPM2-/- n=13, TRPM8-/- n=14, TRPV1-/- n=11, TRPV3-/- n=13, TRPV4-/- n=15). All error bars represent standard errors of the mean. (C) Comparison of Spent time of wild type and TRPM8-/- mice at 11.5 oC in 60 min.

**Additional file 3: Figure S3**. “Spent time” under the condition from 19.7 ℃ to 35.3 ℃. (A) “Spent time” for all genotypes at each temperature zone under the condition of 19.7 ℃ to 35.3 ℃ in a 60-min experiment (wild type n=12, TRPA1-/- n=14, TRPM2-/- n=12, TRPAM8-/- n=12, TRPV1-/- n=11, TRPV3-/- n=13, TRPV4-/- n=15). (B) “Spent time” for all genotypes at each temperature zone under the condition of 19.7 ℃ to 35.3 ℃ across 60 min, as broken down by the first (top), middle (middle), and last (bottom) 20 min. Error bars represent standard errors of the mean. (C) Comparison of Spent time of wild type and TRPA1-/- mice at 33.9 oC in the 40–60 min period.

**Additional file 4: Figure S4**. “Travel distance” under the condition of 11.5 ℃ to 43.6 ℃. (A) “Travel distance” of all genotypes without a thermal gradient across a 60-min experiment (wild type n=12, TRPA1-/- n=14, TRPM2-/- n=12, TRPAM8-/- n=12, TRPV1-/- n=11, TRPV3-/- n=13, TRPV4-/- n=12). (B) Transition of “Travel distance” across whole temperature zones for each genotype in the first, middle, and last 20 min of an experiment. The color of each genotype is the same as shown in panel A. Mice were classified into two groups, as shown by the brackets. * p < 0.05, ** p < 0.01 vs. Wild type. ns not significant. (C) “Travel distance” of all genotypes at each temperature zone from 11.5 ℃ to 43.6 ℃ across the 60 min, as broken down by the first (top), middle (middle), and last (bottom) 20 min of the experiment. Error bars represent standard errors of the mean.

**Additional file 5: Figure S5**. “Moving speed” under the condition of a 11.5 ℃ to 43.6 ℃ gradient. (A) “Moving speed” of all genotypes without a thermal gradient across a 60-min experiment (wild type n=12, TRPA1-/- n=14, TRPM2-/- n=12, TRPAM8-/- n=12, TRPV1-/- n=11, TRPV3-/- n=13, TRPV4-/- n=12). (B) Transition of “Moving speed” across all temperature zones for each genotype in the first, middle, and last 20-min interval of a 60-min experiment. The color of each genotype is the same as in panel A. Mice were classified into two groups, as shown by the brackets. (C) “Moving speed” of all genotypes at each temperature zone under the condition from 11.5 ℃ to 43.6 ℃ across a 60-min experiment, as broken down by the first (top), middle (middle), and last (bottom) 20 min. All error bars represent standard errors of the mean.

## Data Availability

All the data and materials are available at the website of The Journal of Physiological Sciences.

## Abbreviations

LPB: Lateral parabrachial nucleus
MnPO: Median POA
POA: Preoptic area
TRP: Transient receptor potential
WT: Wild type
