# Supplementary figures and images for: Thermal gradient ring reveals different temperature-dependent behaviors in mice lacking thermosensitive TRP channels
Source: J Physiol Sci. 2022 May 26;72:11. doi: 10.1186/s12576-022-00835-3 (PMC10717490; doi:10.1186/s12576-022-00835-3)

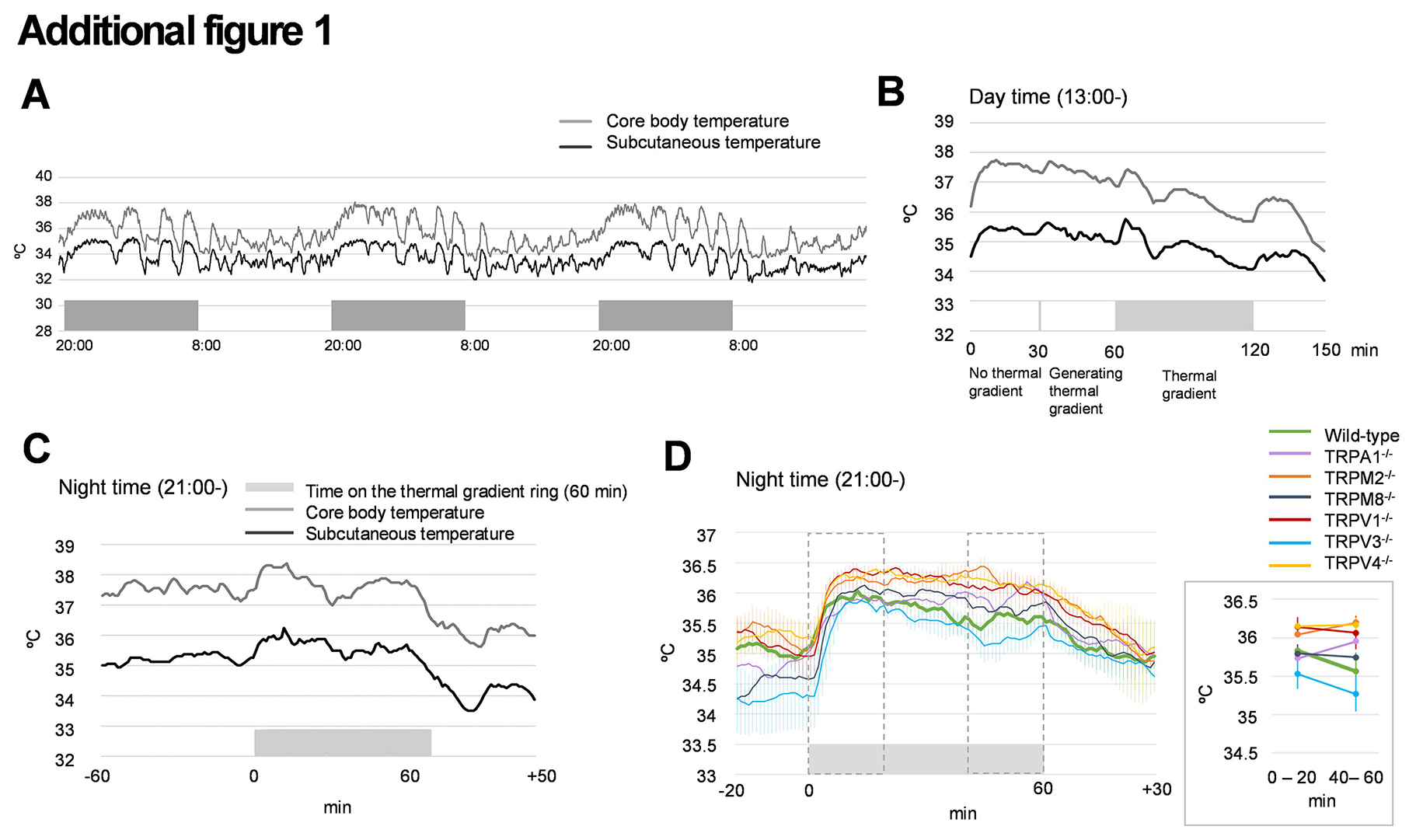

Supplement: Supplementary file 1 — Additional file 1: Figure S1. Changes in body temperatures. (A) Transition of subcutaneous (black) and core (gray) temperatures of WT mice in three consecutive days in the home cage. Nighttime (when the light is turned off from 20:00 to 8:00) is indicated by gray bars. (B) Transition of subcutaneous (black) and core (gray) temperatures of WT mice on the Thermal Gradient Ring during the daytime (13:00–18:00). The Thermal Gradient Ring system is turned on at the 30-min mark of the mouse being placed in the apparatus and the ring floor temperatures are considered stable at 60 to 120 min (shown in gray). (C) Transition of subcutaneous (black) and core (gray) body temperatures before, during (a gray bar), and after a thermal behavior test during the nighttime (21:00–6:00). (D) Transition of subcutaneous temperatures before, during (a gray bar), and after a thermal behavior test in wild-type mice and in mice lacking TRP channels during the nighttime (21:00-) (n=3/genotype). Averaged subcutaneous temperatures of all genotypes in the first and last 20 min are shown in the gray box. Error bars represent standard errors of the mean. [file 12576_2022_835_MOESM1_ESM.jpg]

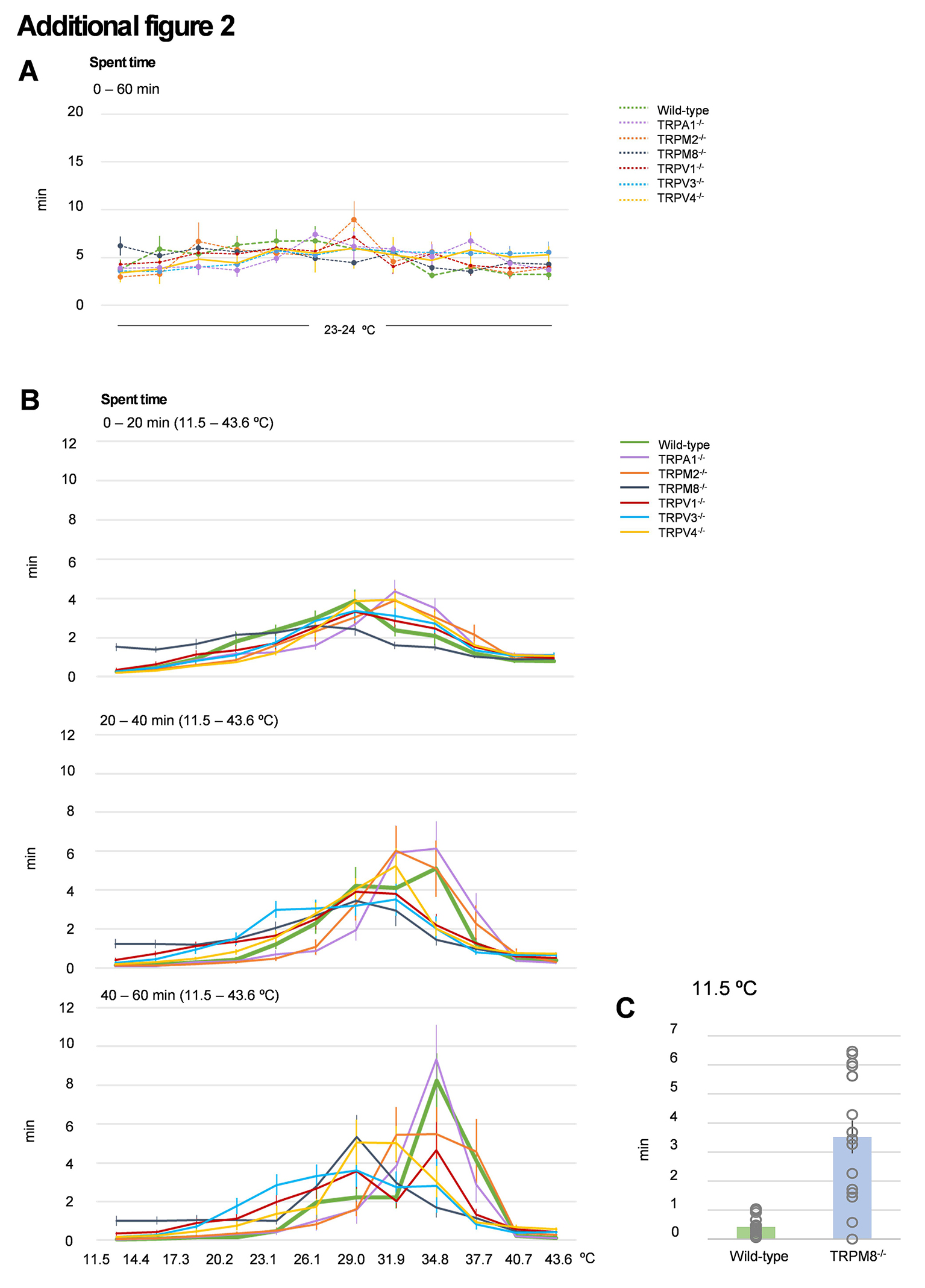

Supplement: Supplementary file 2 — Additional file 2: Figure S2. ‘‘Spent time’’ in the condition from 11.5 to 43.6 ℃. (A) “Spent time” for all genotypes without a thermal gradient across a 60-min experiment (wild type n=12, TRPA1-/- n=14, TRPM2-/- n=12, TRPAM8-/- n=12, TRPV1-/- n=11, TRPV3-/- n=13, TRPV4-/- n=12). (B) “Spent time” for all genotypes at each temperature zone from 11.5 ℃ to 43.6 ℃ across 60 min, as broken down by the first (top), middle (middle), and last (bottom) 20 min of the experiment. (wild type n=15, TRPA1-/- n=14, TRPM2-/- n=13, TRPM8-/- n=14, TRPV1-/- n=11, TRPV3-/- n=13, TRPV4-/- n=15). All error bars represent standard errors of the mean. (C) Comparison of Spent time of wild type and TRPM8-/- mice at 11.5 oC in 60 min. [file 12576_2022_835_MOESM2_ESM.jpg]

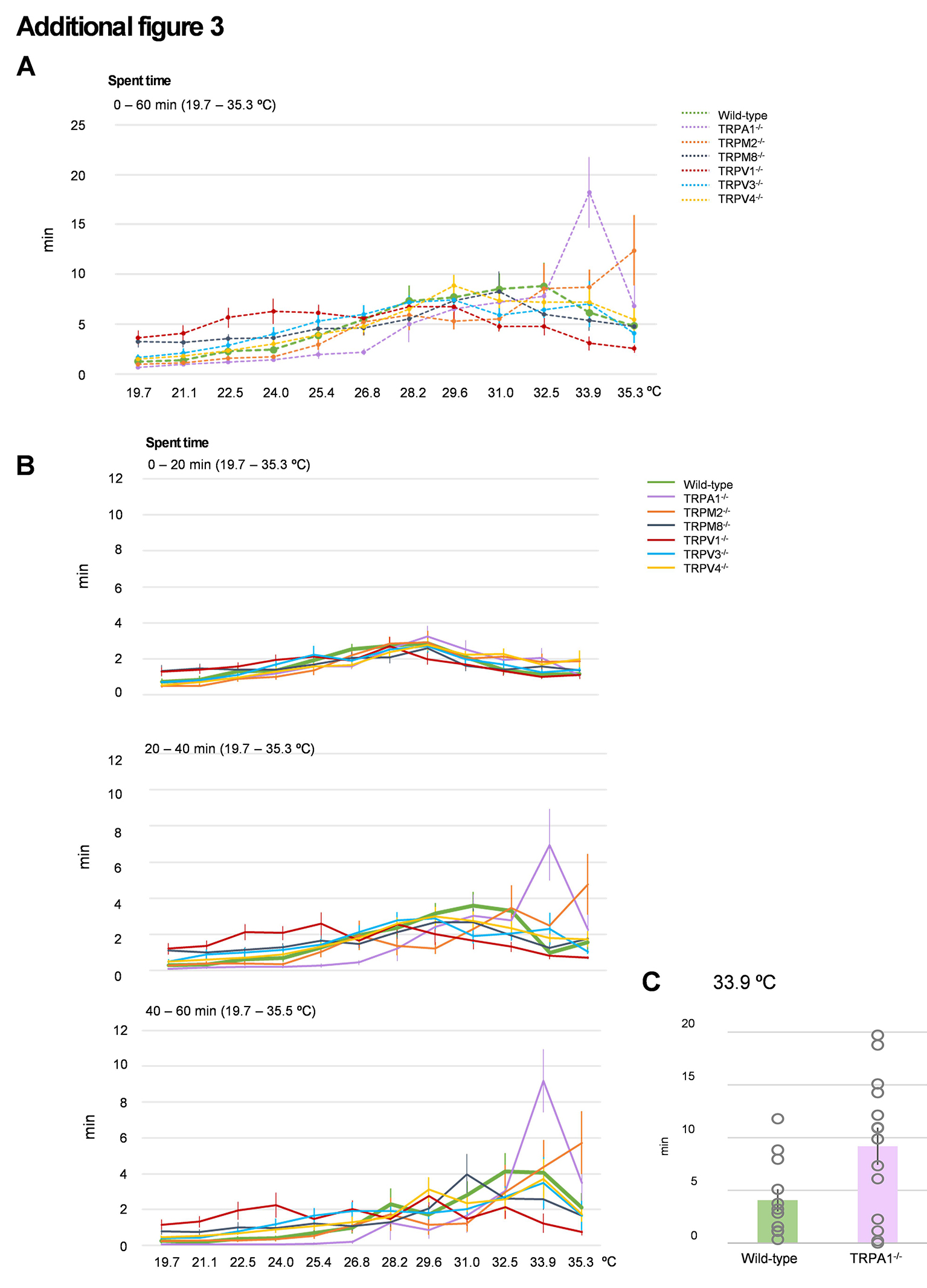

Supplement: Supplementary file 3 — Additional file 3: Figure S3. “Spent time” under the condition from 19.7 ℃ to 35.3 ℃. (A) “Spent time” for all genotypes at each temperature zone under the condition of 19.7 ℃ to 35.3 ℃ in a 60-min experiment (wild type n=12, TRPA1-/- n=14, TRPM2-/- n=12, TRPAM8-/- n=12, TRPV1-/- n=11, TRPV3-/- n=13, TRPV4-/- n=15). (B) “Spent time” for all genotypes at each temperature zone under the condition of 19.7 ℃ to 35.3 ℃ across 60 min, as broken down by the first (top), middle (middle), and last (bottom) 20 min. Error bars represent standard errors of the mean. (C) Comparison of Spent time of wild type and TRPA1-/- mice at 33.9 oC in the 40–60 min period. [file 12576_2022_835_MOESM3_ESM.jpg]

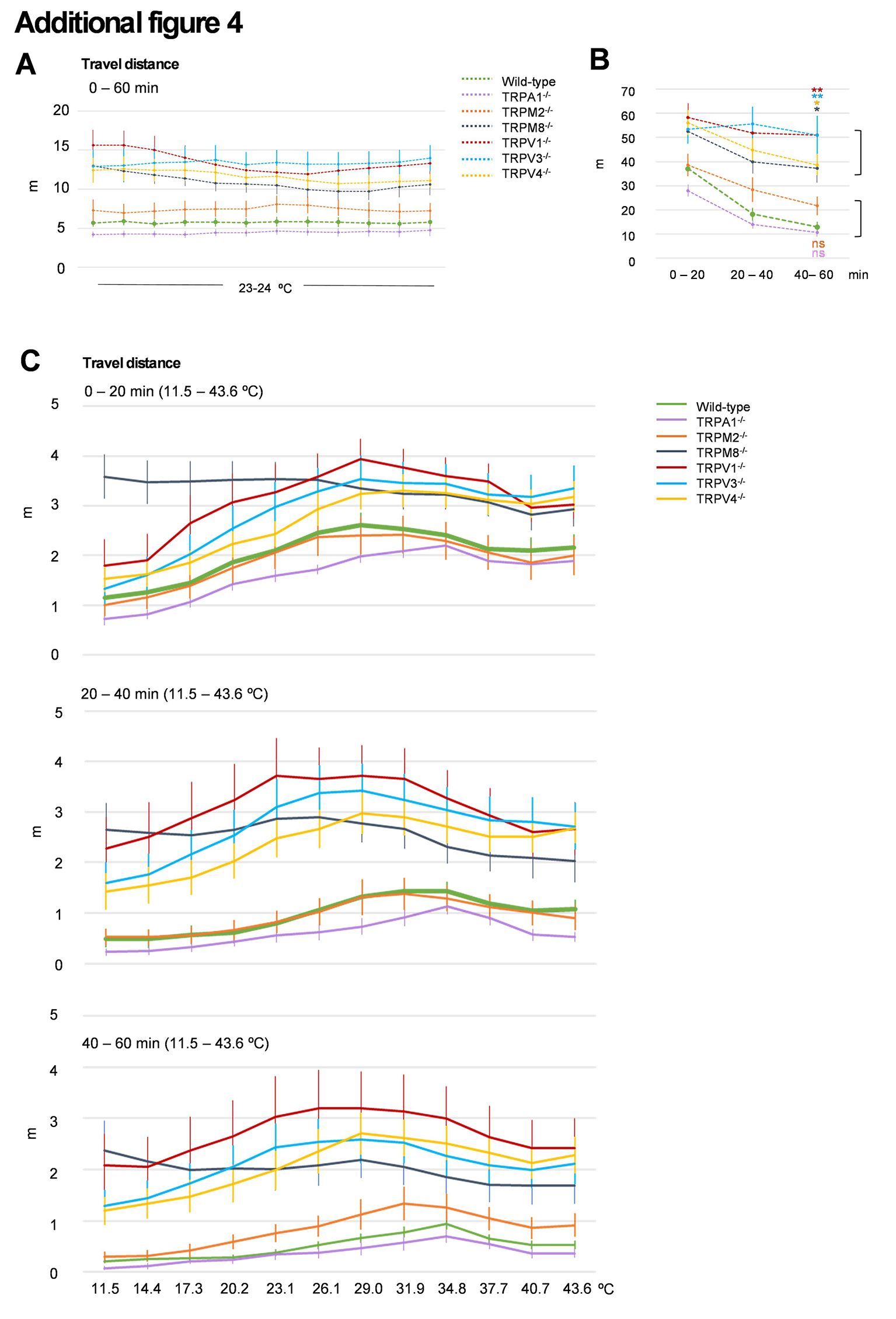

Supplement: Supplementary file 4 — Additional file 4: Figure S4. “Travel distance” under the condition of 11.5 ℃ to 43.6 ℃. (A) “Travel distance” of all genotypes without a thermal gradient across a 60-min experiment (wild type n=12, TRPA1-/- n=14, TRPM2-/- n=12, TRPAM8-/- n=12, TRPV1-/- n=11, TRPV3-/- n=13, TRPV4-/- n=12). (B) Transition of “Travel distance” across whole temperature zones for each genotype in the first, middle, and last 20 min of an experiment. The color of each genotype is the same as shown in panel A. Mice were classified into two groups, as shown by the brackets. * p < 0.05, ** p < 0.01 vs. Wild type. ns not significant. (C) “Travel distance” of all genotypes at each temperature zone from 11.5 ℃ to 43.6 ℃ across the 60 min, as broken down by the first (top), middle (middle), and last (bottom) 20 min of the experiment. Error bars represent standard errors of the mean. [file 12576_2022_835_MOESM4_ESM.jpg]

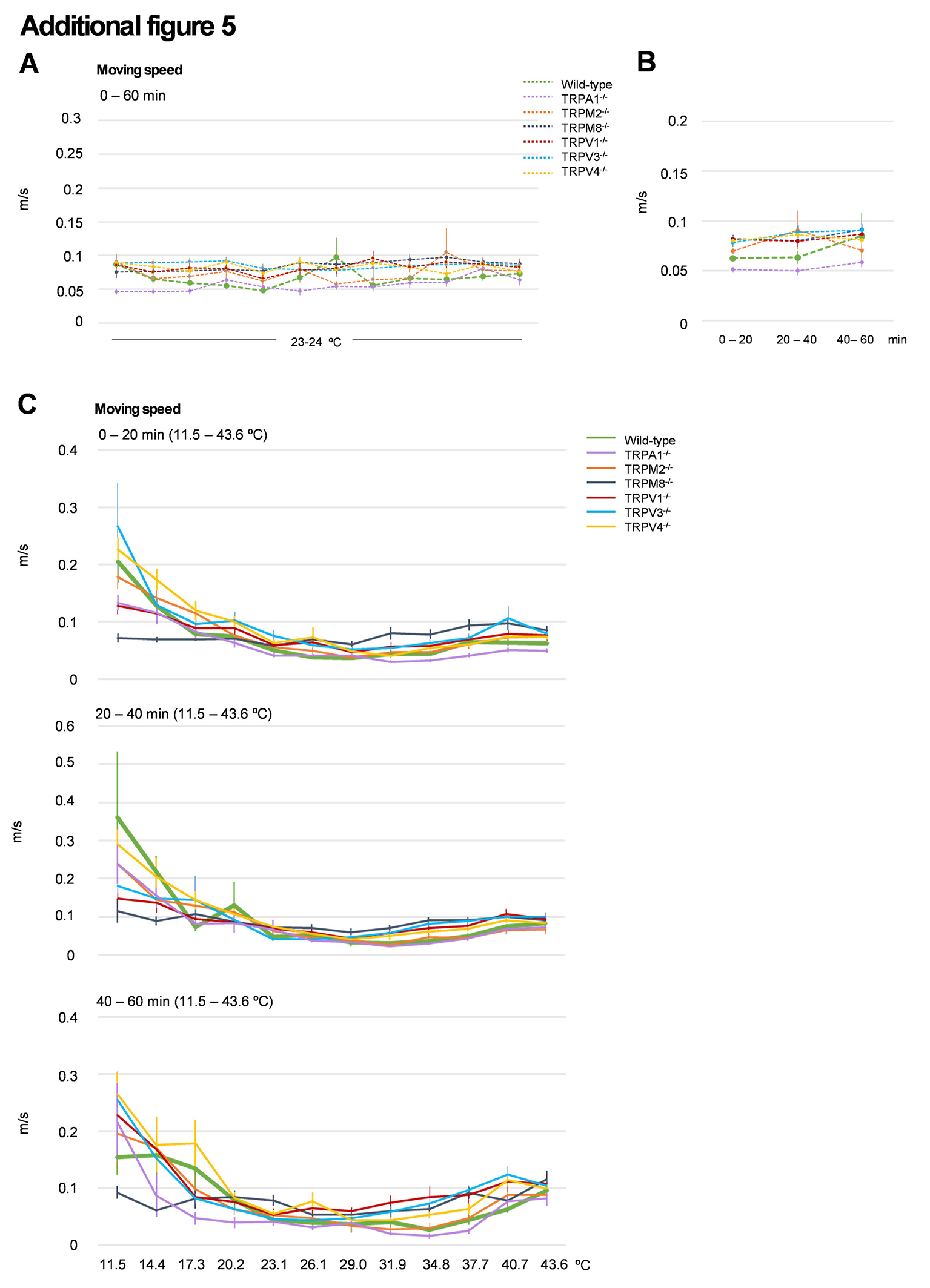

Supplement: Supplementary file 5 — Additional file 5: Figure S5. “Moving speed” under the condition of a 11.5 ℃ to 43.6 ℃ gradient. (A) “Moving speed” of all genotypes without a thermal gradient across a 60-min experiment (wild type n=12, TRPA1-/- n=14, TRPM2-/- n=12, TRPAM8-/- n=12, TRPV1-/- n=11, TRPV3-/- n=13, TRPV4-/- n=12). (B) Transition of “Moving speed” across all temperature zones for each genotype in the first, middle, and last 20-min interval of a 60-min experiment. The color of each genotype is the same as in panel A. Mice were classified into two groups, as shown by the brackets. (C) “Moving speed” of all genotypes at each temperature zone under the condition from 11.5 ℃ to 43.6 ℃ across a 60-min experiment, as broken down by the first (top), middle (middle), and last (bottom) 20 min. All error bars represent standard errors of the mean. [file 12576_2022_835_MOESM5_ESM.jpg]
